# A practical *O*(*n *log^2 ^*n*) time algorithm for computing the triplet distance on binary trees

**DOI:** 10.1186/1471-2105-14-S2-S18

**Published:** 2013-01-21

**Authors:** Andreas Sand, Gerth Stølting Brodal, Rolf Fagerberg, Christian NS Pedersen, Thomas Mailund

**Affiliations:** 1 Bioinformatics Research Center, Aarhus University, Denmark; 2 Department of Computer Science, Aarhus University, Denmark; 3 MADALGO, Center for Massive Data Algorithms, a Center of the Danish National Research Foundation, Denmark; 4 Department of Mathematics and Computer Science, University of Southern Denmark, Denmark; 5 PUMPKIN, Center for Membrane Pumps in Cells and Disease, a Center of the Danish National Research Foundation, Denmark

## Abstract

The triplet distance is a distance measure that compares two rooted trees on the same set of leaves by enumerating all sub-sets of three leaves and counting how often the induced topologies of the tree are equal or different. We present an algorithm that computes the triplet distance between two rooted binary trees in time *O *(*n *log^2 ^*n*). The algorithm is related to an algorithm for computing the quartet distance between two unrooted binary trees in time *O *(*n *log *n*). While the quartet distance algorithm has a very severe overhead in the asymptotic time complexity that makes it impractical compared to *O *(*n*^2^) time algorithms, we show through experiments that the triplet distance algorithm can be implemented to give a competitive wall-time running time.

## Background

Using trees to represent relationships is widespread in many scientific fields, in particular in biology where trees are used e.g. to represent species relationships, so called phylogenies, the relationship between genes in gene families or for hierarchical clustering of high-throughput experimental data. Common for these applications is that differences in the data used for constructing the trees, or differences in the computational approach for constructing the trees, can lead to slightly different trees on the same set of leaf IDs.

To compare such trees, distance measures are often used. Common distance measures include the Robinson-Foulds distance [1], the triplet distance [2], and the quartet distance [3]. Common for these three distance measures is that they all enumerate certain features of the trees they compare and count how often the features differ between the two trees. The Robinson-Foulds distance enumerates all edges in the trees and tests if the bipartition they induce is found in both trees. The triplet distance (for rooted trees) and quartet distance (for unrooted trees) enumerate all subsets of leaves of size three and four, respectively, and test if the induced topology of the leaves is the same in the two trees.

Efficient algorithms to compute these three distance measures exist. The Robinson-Foulds distance can be computed in time *O *(*n*) [4] for trees with *n *leaves, which is optimal. The quartet distance can be computed in time *O *(*n *log *n*) for binary trees [5], in time *O *(*d*^9 ^*n *log *n*) for trees where all nodes have degree less than *d *[6], and in sub-cubic time for general trees [7]. See also Christiansen *et al. *[8] for a number of algorithms for general trees with different tradeoffs depending on the degree of inner nodes. For the triplet distance, *O *(*n*^2^) time algorithms exist for both binary and general trees [2,9].

Brodal *et al. *[5] present two algorithms for computing the quartet distance for binary trees; one running in time *O *(*n *log *n*), and one running in time *O *(*n *log^2 ^*n*). The latter is the most practical, and was implemented in [10,11], where it was shown to be slower in practice compared to a simple *O *(*n*^2^) time algorithm [12] unless *n *is above 2000. In this paper we focus on the triplet distance and develop an *O *(*n *log^2 ^*n*) time algorithm for computing this distance between two rooted binary trees. The algorithm is related to the *O *(*n *log^2 ^*n*) time algorithm for quartet distance, but its core accounting system is completely changed. As we demonstrate by experiments, the resulting algorithm is not just theoretically efficient but also Efficient in practice, as it is faster than a simple *O *(*n*^2^) time algorithm based on [12] already for *n *larger than 12, and is e.g. faster by a factor of 50 when *n *is 2900.

## Methods

**Figure 1:**
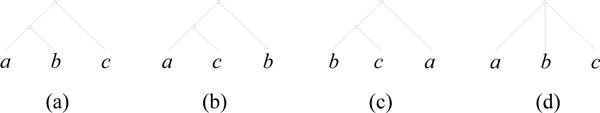
**Triplet topologies**. The four different triplet toplogies.

The triplet distance measure between two rooted trees with the same set of leaf IDs is based on the topologies induced by a tree when selecting three leafs of the tree. Whenever three leaves, *a, b *and *c*, are selected, a tree can induce one of four topologies: It can either group *a *and *b, a *and *c*, or *b *and *c*, or it can put them at equal distance to the root, i.e. all three pairs of leaves have the same lowest common ancestor (see Figure 1). For binary trees, the last case is not possible since this would require a node with at least three children.

The triplet distance is the number of triplets whose topology differ in the two trees. It can naïvely be computed by enumerating all *O *(*n*^3^) sets of three leafs and comparing the induced topologies in the two trees, counting how often the trees agree or disagree on the topology. Triplet topologies in a tree, however, are not independent, and faster algorithms can be constructed exploiting this, comparing sets of triplet topologies faster. Critchlow *et al. *[2] for example exploit information about the depth of shared ancestors of leaves in a tree to achieve an *O *(*n*^2^) time algorithm for binary trees while Bansal *et al. *[9] construct a table of shared leaf-sets and achieve an *O *(*n*^2^) time algorithm for general trees.

For the quartet distance, the analogue to the triplet distance for unrooted trees, Brodal *et al. *[5] construct an even faster algorithm by identifying sets of four different leaves in one tree through coloring all leaves with one of three different colors and then counting the number of topologies compatible with the coloring in the other tree. Using a variant of the so-called "smaller half trick" for keeping the number of different relevant colorings low, the algorithm manages to construct all relevant colorings with *O *(*n *log *n*) color changes. The number of topologies compatible with the coloring can then be counted in the other tree using a data structure called a "hierarchical decomposition tree". Maintaining the hierarchical decomposition tree, however, involves a number of polynomial manipulations that, while theoretically can be done in constant time per polynomial, are quite time consuming in practice [10,11], making the algorithm slow in practice.

A naïve algorithm that computes the quartet distance between two unrooted trees by explicitly inspecting each of the *O *(*n*^4^) quartets can be modified to compute the triplet distance between two rooted trees without loss of time by adding a new leaf *x *above the two root nodes and limit the inspection of quartets to the quartets containing this new leaf. However, the efficient algorithms for computing the quartet distance presented in [5,7] do not explicitly inspect every quartet and therefore cannot be modified to compute the triplet distance following this simple approach.

In the following, we develop an efficient algorithm for computing the triplet distance between two rooted binary trees *T*_1 _and *T*_2 _with the same set of leaf IDs. Our key contribution is to show how all triplets in one tree, say *T*_1_, can be captured by coloring the leaves with colors, and how the smaller half trick lets us enumerate all such colorings in time *O *(*n *log *n*). We will then construct a hierarchical decomposition tree (HDT) for *T*_2 _that counts its number of compatible triplets. Unlike the algorithms for computing the quartet distance [5], where the counting involves manipulations of polynomials, the HDT for triplets involves simple arithmetic computations that are efficient in practice.

### Counting shared triplets through leaf colorings

A triplet is a set {*a, b, c*} of three leaf IDs. For a tree *T*, we assign each of the $\left(\begin{array}{c} n\\ 3 \end{array}\right)$ triplets to the lowest common ancestor in *T *of the three leaves containing *a, b*, and *c*. For a node *v *∈ *T *we denote by *τ_v _*the set of triplets assigned to *υ*. Then {*τ_v _| v *∈ *T*} is a partition of the set T  of triplets. Thus, {*τ_v _∩ τ_u _| v T*_1_, *u *∈ *T*_2_} is also a partition of T . Our algorithm will find

$$\text{Shared}\left(T\right)=\underset{v\in T_{1}}{\sum }\underset{u\in T_{2}}{\sum }\text{Shared}\left(\tau_{v}\cap \tau_{u}\right),$$

where Shared(*S*) on a set *S *of triplets is its number of triplets having the same topology in *T*_1 _and *T*_2_. The triplet distance of *T*_1 _and *T*_2 _is then $\left(\begin{array}{c} n\\ 3 \end{array}\right)-\text{Shared}\left(T\right)$.

**Figure 2:**
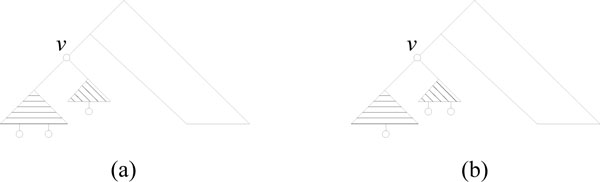
**Coloring when visiting a node**. Coloring of a sub-tree rooted in node *v *lets us count all triplets rooted in *v*.

In the algorithm, we capture the triplets *τ_v _*by a coloring of the leaves: if all leaves not in the subtree of *v *have no color, all leaves in one subtree of *v *are "red", and all leaves in the other subtree are "blue", then *τ_v _*is exactly the triplets having two leaves colored "red" and one leaf colored "blue", or two leaves colored "blue" and one leaf colored "red". See Figure 2.

For such a coloring according to a node *v *∈ *T*_1_, and for a node *u *∈ *T*_2_, the number Shared(*τ_v _∩ τ_u _*) could be found as follows: let *x *and *y *be the two subtrees of *u*, let *x*(*r*) and *y*(*r*) be the number of leaves colored red in the subtrees *x *and *y*, respectively, and *x*(*b*) and *y*(*b*) the number of leaves colored blue. The number Shared(*τ_v _∩ τ_u _*) is then $\left(\begin{array}{c} x\left(b\right)\\ 2 \end{array}\right)\;\cdot \;y\left(r\right)\;+\;\left(\begin{array}{c} y\left(b\right)\\ 2 \end{array}\right)\;\cdot \;x\left(r\right)+\left(\begin{array}{c} y\left(r\right)\\ 2 \end{array}\right)\;\cdot \;x\left(b\right)\;+\;\left(\begin{array}{c} x\left(r\right)\\ 2 \end{array}\right)\;\cdot \;y\left(b\right).$ We call these the triples of *u *compatible with the coloring.

Explicitly going through *T*_1 _and coloring for each node *v *would take time *O *(*n*) per node, for a total time of *O *(*n*^2^). We reduce this to *O *(*n *log *n*) by using the smaller half trick. Going through *T*_2 _for each coloring and counting the number of compatible triplets would also take time *O *(*n*^2^). Using a HDT we find this count in *O*(1) time, while updating the structure takes time *O *(log *n*) after each leaf color change. The result is *O*(*n *log^2 ^*n*) total running time. In essence, the HDT performs the inner sum of $\text{Shared}\left(T\right)=\sum_{v\in T_{1}}\sum_{u\in T_{2}}\text{Shared}\left(\tau_{v}\cap \tau_{u}\right)$, while the coloring algorithm performs the outer sum.

### Smaller half trick

We go through nodes *v *in a depth first order while maintaining two invariants of the algorithm: 1) Before we process *v*, the entire subtree of *v *is colored "red" and the rest of the tree has no color; 2) When we return from the depth first recursion, the entire tree has no color.

**Figure 3:**
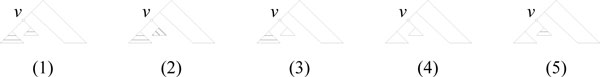
**Coloring algorithm**. The five steps of the coloring in the smaller-half trick.

For *v *let *S*(*v*) denote the smallest subtree of *v *and let *L*(*v*) denote the largest subtree of *v*. We go through the coloring as follows (see Figure 3):

1. Color *S*(*v*) "blue". Now *v *has the coloring that enable us to count the triplets for *v*.

2. Remove the color for *S*(*v*). Now we can call recursively on *L*(*v*) while satisfying the invariant.

3. Returning from the recursive call, the entire tree is colorless by invariant 2.

4. Color *S*(*v*) "red". Now we satisfy invariant 1 for calling recursively on *S*(*v*).

5. Call recursively on *S*(*v*). When we return we satisfy invariant 2 for returning from the recursive call.

Using this recursive algorithm, we go through all colorings of the tree. In each instance (not counting recursive calls), we only color leaves in *S*(*v*), and only a constant number of times. Thus, a leaf ℓ is only colored when visiting an ancestor node *v *where ℓ *S*(*v*), i.e. ℓ is in the smaller subtree of *v*. Since ℓ can have at most *O *(log *n*) such ancestors, each leaf will only be colored at most *O *(log *n*) times, implying a total of *O *(*n *log *n*) color changes.

### Hierarchical decomposition tree

We build a data structure, the *hierarchical decomposition tree *(HDT), on top of the second tree *T*_2 _in order to count the triplets in *T*_2 _compatible with the coloring of leaves in the first tree *T*_1_. The HDT is a balanced binary tree where each node corresponds to a connected part of *T*_2_. Each node in the HDT, or *component*, keeps a count of the number of compatible triplets the correspondent part of *T*_2 _contains, plus some additional book-keeping that makes it possible to compute this count in each component in constant time using the information stored in the component's children in the HDT.

The HDT contains three different kinds of components:

• **L**: A leaf in *T*_2_,

• **I**: An inner node in *T*_2_,

• **C**: A connected sub-part of *T*_2_,

where for type **C **we require that at most two edges in *T*_2 _crosses the boundary of the component; at most one going up towards the root and at most one going down to a subtree.

**Figure 4:**
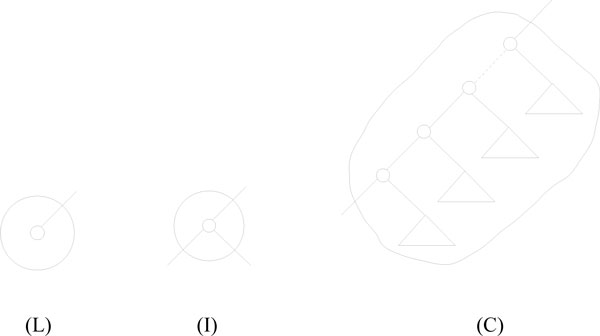
**Component types in the HDT**. The three different types of components. **L **and **I **components contain a single node from the underlying tree while **C **components contain a connected set of nodes.

The leaves and inner nodes of *T*_2 _are transformed into **L **and **I **components, respectively, and constitute the leaves of the HDT. **C **components are then formed by pairwise joining other components along an edge in *T*_2 _by one of two compositions, see Figure 4. **C **components can be thought of as consisting of a path from a sub-tree below the **C **component going up towards the root of *T*_2_, such that all trees branching o to other children along the path are all contained in the component. In the following we show how the HDT of *T*_2 _can be constructed in time *O*(*n*), and we prove that the height of the HDT is *O *(log *n*).

The construction algorithm operates on *components *and *edges*. Each component is of one of the types **L, I**, or **C**. It has a *parent *pointer, pointing to its parent component in the HDT, and a bit, *down_closed*, indicating if the component corresponds to a complete subtree in the original tree. When a component does not yet have a parent in the HDT, we make the *parent *pointer point to the component itself. We use this pointer to test if an edge has been contracted in the HDT construction. Edges consist of two pointers, an *up *pointer and a *down *pointer that points to the components above and below the edge in *T*_2_.

**Figure 5:**
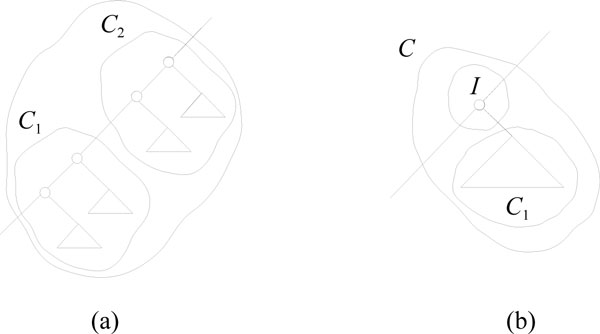
**Component compostions in the construction of the HDT**. The two different ways of constructing a **C **component by merging two underlying components. The topmost of the components can either be a **C **component (a) or an **I **component (b) while the bottommost component, *C*_1_, must be a **C **or **L **component. If the topmost component is an **I **component, the bottommost must be downwards closed, i.e. it cannot have a downwards edge crossing its boundary.

**Figure 6:**
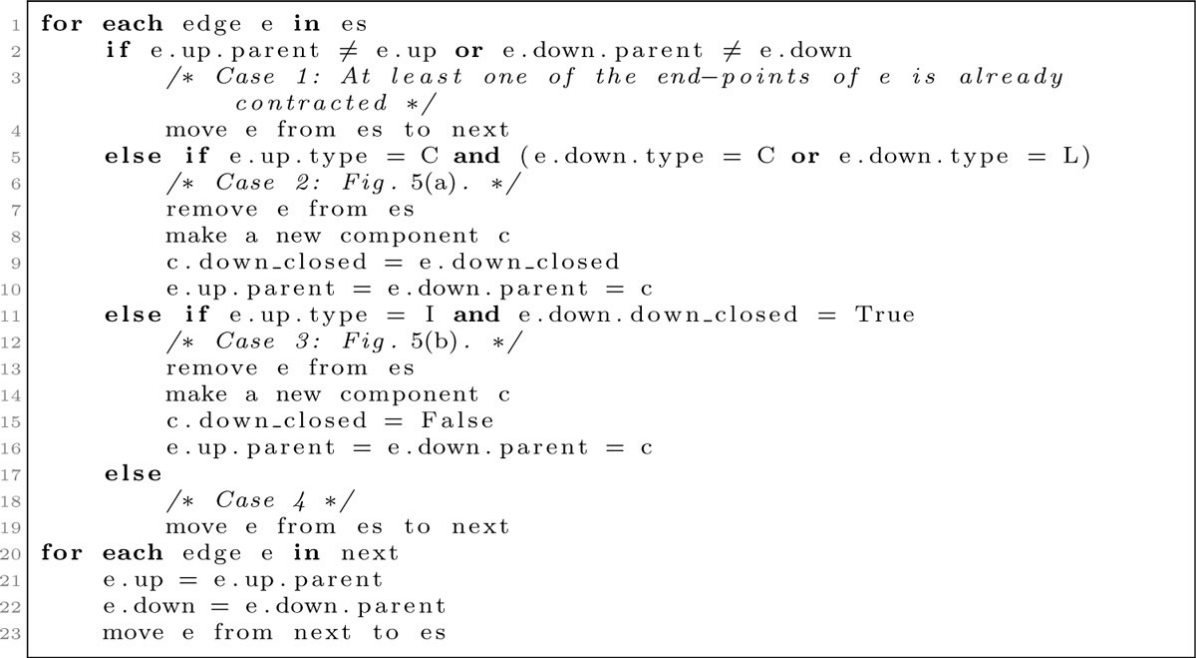
**Algorithm for constructing the hierarchical decomposition tree**. Algorithm for constructing the hierarchical decomposition tree. The listing shows the algorithm run for each level of the HDT construction. This algorithm is repeated until the *es *list is empty.

In a single traversal of the tree, the algorithm initially builds a component for each node in the tree (an **L **component for each leaf and an **I **component for each inner node) and an edge for each edge in the tree. The *parent *pointer of each component is initially set to point to the component itself, and the *down_closed *bit is set to true for **L **components and false for **I **components. The edges are put in a list *es*. We then perform a series of iterations, each constructing one level of the HDT. In each iteration, edges whose neighbors can be joined to form a **C **component via the constructions in Figure 5 are greedily removed from *es*, and another list, *next*, is used to keep the edges that cannot be contracted in this iteration. The details of an iteration is shown in Figure 6. The process stops when *es *becomes empty.

In case 1, one of *e's *neighbors already has a parent, thus this neighbor has already been contracted into a **C **component in this iteration and should not be contracted again. Case 2 is the situation in Figure 5a, and case 3 is the situation in Figure 5b. In case 4, *e.down *is an **I **component or *e.up *is an **I **component and *e.down *does not correspond to a complete subtree, hence none of the constructions in Figure 5 apply. After removing all edges from *es*, the *up *and *down *pointers of the remaining edges in *next *are updated in lines 21-22, such that they point to either a newly created component or still point to the same component. The edges in *next *are finally moved back to *es *in line 23, and a new iteration is started. The algorithm finishes when *es *is empty, and the root of the HDT is the **C **component resulting from joining the ends of the last edge. We now argue that height of the HDT is *O*(log *n*), and that the construction time is *O*(*n*). Each iteration of the code in Figure 6 takes time linear in the number *E *= *|es| *of edges remaining to be contracted at the beginning of the iteration. The height and time bounds follow if we can argue that the number of edges decreases geometrically for each iteration. In the following we argue that the number of edges after one iteration is at most 11/12. *E*.

We first argue that the number of contractible edges at the beginning of the iteration is at least *E*/4. Note that only edges incident to **I **components might not be contractible, and that the number of down-closed components is at least one larger than the number of **I **components. If the number of **I **components is at most *E*/4, then at most 3 · *E*/4 incident edges might not be contractible, i.e. at least *E*/4 edges are contractible. Otherwise the number of **I **components is more than *E*/4, and therefore the number of down-closed components is more than *E*/4 + 1. Since the parent edges from all down-closed components are contractible (for *E *≥ 1), the number of contactable edges is again at least *E*/4.

Since each contracted edge can prevent at most two other edges incident to the two merged components (see Figure 5) from being contracted in the same iteration, each iteration will contract at least 1/3 of the at least *E*/4 contractible edges. It follows that an iteration reduces the number of contractible edges by at least *E*/12.

### Counting triplets in the hierarchical decomposition tree

In each component we keep track of *N*, the number of triplets contained within the component (i.e., where the three leaves of the triplet are within the component) that are compatible with the coloring. When we change the coloring, we update the HDT to reflect this, so we can always read o the total number of compatible triplets from the root of the HDT in constant time.

By adding a little book-keeping information to each component we make it possible to compute *N *(and the book-keeping information itself) for a component in constant time from the information in the component's children in the HDT. This has two consequences: it makes it possible to add the book-keeping and *N *to all components after the construction in linear time, and it makes it possible to update the information when a leaf changes color by updating only components on the path from the leaf-component to the root in the HDT, a path that is bounded in length by *O *(log *n*). Since we only change the color of a leaf *O *(*n *log *n*) times in the triplet distance algorithm, it is this property of the HDT that keeps the total running time at *O *(*n *log^2 ^*n*). For the book-keeping we store six numbers in each component in addition to *N*:

• *R*: The number of leaves colored red in the component.

• *B*: The number of leaves colored blue in the component.

**Figure 7:**
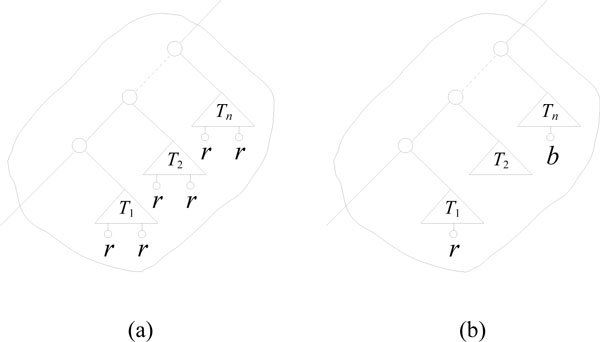
**Counting triplets in a C component**. The two cases of counting triplets in a **C **component.

• $\tilde{rr}$: The number of pairs of red leaves found in the same sub-tree of a **C **component. Let *T_i_, i *= 1,..., *n*, denote the sub-trees in the component, see Figure 7a, and let *r*(*i*) denote the number of red leaves in tree *T_i_*. Then $\tilde{rr}=\sum_{i=1}^{n}\left(\begin{array}{c} r\left(i\right)\\ 2 \end{array}\right)$.

• $\tilde{bb}$: The number of pairs of blue leaves found in the same sub-tree of a **C **component.

• $\hat{rb}$: The number of pairs of leaves with a red leaf in one sub-tree and a blue in another sub-tree, where the red leaf is in a tree further down on the path in a **C **component. Let *T_i_, i *= 1,..., *n*, denote the sub-trees in the component, see Figure 7, and let *r*(*i*) denote the number of red leaves in tree *T_i _*and *b*(*i*) the number of blue leaves in *T_i_*. Then $\hat{rb}=\sum_{i=1}^{n}\sum_{j=i+1}^{n}r\left(i\right)\;\cdot \;b\left(j\right)$.

• $\hat{br}$: The number of pairs of leaves with a red leaf in one sub-tree and a blue in another sub-tree, where the blue leaf is in a tree further down on the path in a **C **component.

We describe how the book-keeping variables and *N *are computed through a case-analysis on how the components are constructed. **L **and **I **components are constructed in only one way, while **C **components are constructed in one of two ways (see Figure 5).

**L components: **For a leaf component, *R *is 1 if the leaf is colored red and 0 otherwise, *B *is 1 if the leaf is colored blue and 0 otherwise, and all other counts are 0.

**I components: **All counts are 0.

**C components, case Figure **5a: Let *x *be one of the counters *R, B*, $\tilde{rr}$,... listed above for a **C **component, and let *x*(1) and *x*(2) denote the corresponding counter in component *C*_1 _and *C*_2_, respectively, with *C*_2 _above *C*_1 _in the underlying tree. Then

$$\begin{array}{c} R=R\left(1\right)+R\left(2\right)\\ \tilde{rr}=\tilde{rr}\left(1\right)+\tilde{rr}\left(2\right)\\ \hat{rb}=\hat{rb}\left(2\right)+\hat{rb}\left(1\right)+R\left(1\right)\cdot B\left(2\right) \end{array}\;\;\begin{array}{c} B=B\left(1\right)+B\left(2\right)\\ \tilde{bb}=\tilde{bb}\left(1\right)+\tilde{bb}\left(2\right)\\ \hat{br}=\hat{br}\left(2\right)+\hat{br}\left(1\right)+B\left(1\right)\cdot R\left(2\right). \end{array}$$

The triplet count is then computed as

$$\begin{array}{ccc} N & =N\left(1\right)+N\left(2\right)+\left(\begin{array}{c} R\left(1\right)\\ 2 \end{array}\right)\;\cdot \;B\left(2\right)+\left(\begin{array}{c} B\left(1\right)\\ 2 \end{array}\right)\cdot \;R\left(2\right) & \\ \; & +\tilde{rr}\left(2\right)\;\cdot \;B\left(1\right)+\tilde{bb}\left(2\right)\;\cdot \;R\left(1\right)+R\left(1\right)\;\cdot \;\hat{rb}\left(2\right)+B\left(1\right)\;\cdot \;\hat{br}\left(2\right). \end{array}$$

**C components, case Figure **5b: Let *x*(1) denote one of the counters listed above for component *C*_1_. Then *R *= *R*(1), *B *= *B*(1), $\tilde{rr}=\left(\begin{array}{c} R\left(1\right)\\ 2 \end{array}\right)$, $\tilde{bb}=\left(\underset{2}{B(1)}\right)$, $\hat{rb}=\hat{br}=0$. Since the inner node in the composition does not contain any leaves, the triplet count is simply *N *= *N *(1).

## Results and discussion

We implemented the algorithm in C++ and a simple *O*(*n*^2^) time algorithm to ensure that it computes the correct triplet distance.

**Figure 8:**
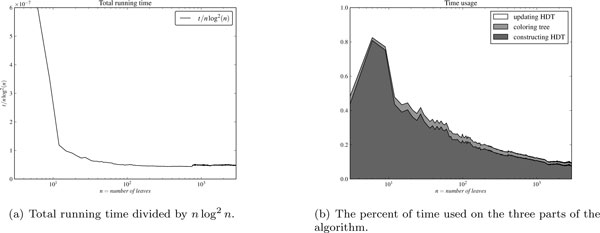
**Validation of running time**. (a) Total running time divided by *n *log^2 ^*n*.. (b) The percent of time used on the three parts of the algorithm. The left figure shows the total running time divided by *n *log^2 ^*n *showing that the theoretical running time is achieved in the implementation. The right figure shows the percent of time used on the three parts of the algorithm: constructing the HDT of the second tree, coloring the leaves in the first tree, and updating the HDT accordingly. Constructing the HDT takes a considerable part of the time, but as the trees grow, updating the HDT takes a larger part. The plots show the average over 50 experiments for each size *n*.

We then verified the running time of our algorithm, see Figure 8a. As seen in the figure, the running time evens out when we divide with *n *log^2 ^*n *giving us confidence that the analyzed running time is correct. We also measured where in the algorithm time was spent, whether it is in constructing the HDT, in coloring the leaves in the first tree, or in updating the counts in the HDT. Figure 8b illustrates the time spend on each of these three parts of the algorithm, normalized so the running time sums to one. For small trees, constructing the HDT makes up a sizable fraction of the running time, not surprising since the overhead in constructing components is larger than updating them. As the size of the trees increase, more time is spent on updating the HDT, as expected since updating the HDT runs in *O *(*n *log^2 ^*n*) while the other operations are asymptotically *O *(*n *log *n*).

When changing the color of leaves, we spent time *O *(log *n*) updating the book-keeping in the HDT for each leaf. We only count after a complete sub-tree has changed color, however, so instead of updating the HDT for each color-change we could just mark which leaves have changed color and then update the HDT bottom-up, so each inner node would only be updated once when it is on a path from a changed leaf. We implemented this, but found that the extra book-keeping from marking nodes and then updating increased the running time by 10%-15% compared to just updating the HDT.

**Figure 9:**
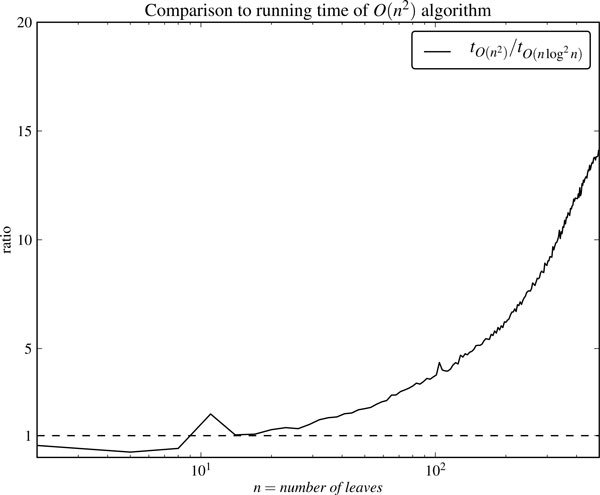
**Comparison to *O*(*n*^2^) algorithm**. Ratio between the running times for the *O*(*n*^2^) time algorithm and the *O*(*n *log^2 ^*n*) time algorithm.

To render the use of the algorithm in practice, we implemented an Efficient *O*(*n*^2^) time algorithm based on the quartet distance algorithm presented in [12]. Figure 9 shows the ratio of the running time for the *O*(*n*^2^) time algorithm against the *O*(*n *log^2 ^*n*) time algorithm. It is evident that our algorithm is fastest for all practical purposes. The speed-up factor for *n *= 2900 is 46.

## Conclusions

We have presented an *O *(*n *log^2 ^*n*) time algorithm for computing the triplet distance between two binary trees and experimentally validated its correctness and time analysis.

The algorithm builds upon the ideas in the *O *(*n *log^2 ^*n*) time algorithm for computing the quartet distance between binary trees [13], but where the book-keeping in the quartet distance algorithm is rather involved, making it inefficient in practice, the book-keeping in the triplet distance algorithm in this paper is entirely different, and significantly simpler and faster.

Compressing the HDT during the algorithm makes it possible to reduce the running time of the quartet distance algorithm to *O *(*n *log *n*) and the same approach can also reduce the running time of the triplet algorithm to *O *(*n *log *n*). We have left for future work to experimentally test whether this method incurs too much overhead to make it practically worthwhile.

Unlike the *O *(*n*^2^) time algorithm of Bansal *et al. *[9] our algorithm does not generalize to non-binary trees. It is possible to extend the algorithm to non-binary trees by employing more colors, as was done for the quartet distance [6], but this makes the algorithm depend on the degree of nodes, and future work is needed to develop a sub-quadratic algorithm for general rooted trees.

## Competing interests

The authors declare that they have no competing interests.

## Authors' contributions

All authors contributed to the design of the presented algorithm. AS implemented the data structures and the algorithm. AS, CNSP, and TM designed the experiments, and AS conducted these. All authors have contributed to, seen and approved the manuscript.

## Declarations

The publication costs for this article were funded by PUMPKIN, Center for Membrane Pumps in Cells and Disease, a Center of the Danish National Research Foundation, Denmark.

This article has been published as part of *BMC Bioinformatics *Volume 14 Supplement 2, 2013: Selected articles from the Eleventh Asia Pacific Bioinformatics Conference (APBC 2013): Bioinformatics. The full contents of the supplement are available online at http://www.biomedcentral.com/bmcbioinformatics/supplements/14/S2.
